# Discrimination of Black and White Sesame Seeds Based on Targeted and Non-Targeted Platforms with Chemometrics: From Profiling towards Identification of Chemical Markers

**DOI:** 10.3390/foods11142042

**Published:** 2022-07-11

**Authors:** Si Mi, Yuhang Wang, Xiangnan Zhang, Yaxin Sang, Xianghong Wang

**Affiliations:** College of Food Science and Technology, Hebei Agricultural University, Baoding 071000, China; misi@hebau.edu.cn (S.M.); yuhangwanghebau@163.com (Y.W.); xiangnanzhanghebau@163.com (X.Z.); yaxinsanghebau@163.com (Y.S.)

**Keywords:** multi-element, volatiles, fatty acids, metabolomics, sesame seeds

## Abstract

The present study was conducted to clarify the differences in the multi-element, volatile organic compound, fatty acid, and metabolite fingerprints between black and white sesame seeds. A total of 53 chemical elements, 32 volatile flavor compounds, 40 fatty acids, and 283 metabolites were identified and evaluated in the two groups of sesame seeds. Univariate and multivariate statistics indicated a distinct separation between the two groups of sesame seeds. A panel of 16 chemical elements, 3 volatile compounds, 8 individual fatty acids, and 54 metabolites with *p* value < 0.05 and variable importance in projection score > 1 were selected as the most important discriminants for the two types of sesame seeds. Overall, these data reveal the influence of genotype on the chemical composition of sesame seeds. Our findings also demonstrate that the hybrid model of instrumental analysis and chemometrics is feasible for the discrimination of black and white sesame seeds.

## 1. Introduction

Sesame is an important oilseed plant belonging to the genus *Sesamum*, and mainly cultivated in tropical and sub-tropical countries [1,2,3]. Sesame seeds contain various nutrients and bioactive compounds, for example, minerals, fatty acids, vitamins (especially E and B group), phenolics, flavonoids, lignans, and other phytochemicals [4,5,6].

Sesame seeds can be utilized as raw materials to produce flour, paste, and other kinds of food products [7,8]. According to the reports, around 65% of the total world sesame seeds are used for the production of edible oil [9]. China is one of the main areas in the world for sesame seed production and processing [2,9,10]. The commercially available products of sesame seeds from the Chinese market can be generally classified into two types, namely, white and black sesame seed-derived products. An interesting phenomenon observed in the marketplace is that black sesame seed-related products are usually sold at a twice or even higher price than that of white sesame seed products. We hypothesize that differences in chemical components may exist between the two types of sesame seeds. 

There have already been a number of research papers concerning the quality evaluation and comparison of different kinds of sesame seeds and derived products. The quality of raw material is strongly associated with the quality of processed products. Sesame seeds, as an important food material, have been investigated in terms of their mineral nutrient contents [5,11], fatty acid composition [8,12], bacterial microbiome [13], phytochemicals [8], volatiles [12], and metabolite composition [2]. The motivations behind these previous studies were to achieve the discrimination of sesame seeds from different geographical origins [2,8,13], or to clarify the effect of agricultural practices or processing on sesame seeds [11,12]. Nevertheless, very few reports are available on the comparison of chemical composition between the two groups of sesame seeds by a combination of multi-element, volatile, fatty acid, and metabolite profiles.

The present study was designed to: (i) establish a relatively comprehensive chemical fingerprint covering multi-element, volatile flavor compounds, fatty acids, and metabolites; and (ii) clarify the differential chemical components present between the two groups of sesame seeds. To achieve these goals, several analytical techniques, namely, inductively coupled plasma-mass spectrometry (ICP-MS), headspace-gas chromatography-ion mobility spectrometry (HS-GC-IMS), gas chromatography-mass spectrometry (GC-MS), and ultra-high performance liquid chromatography with quadrupole time-of-flight mass spectrometry (UHPLC-Q-TOF/MS) were utilized in this research. Furthermore, the discrimination models were constructed by chemometrics methods. 

## 2. Materials and Methods

### 2.1. Chemicals and Standards

LC-grade aqueous ammonia, ammonium formate (>99% purity), methanol, chloroform, and GC-grade n-hexane were provided by Sigma-Aldrich Trading Co, Ltd. (Shanghai, China). Optima LC-MS grade water and acetonitrile were purchased from Merck KGaA (Darmstadt, Germany). The elemental standards investigated in the present research were obtained from Chinese Standard Station (CSS, Beijing, China) and Agilent Technologies (Palo Alto, CA, USA). The authentic standards of 40-component fatty acid methyl ester (FAME) mix and 2-methyl-3-heptanone (IS; >99% purity) were ordered from Sigma-Aldrich Trading Co, Ltd. (Shanghai, China). Methyl salicylate (GC; >99% purity) was purchased from TCI Development Co., Ltd. (Shanghai, China).

### 2.2. Sample Information and Treatment

Sesame seed samples were obtained from Zhongao Food Co., Ltd. located in Handan, China. They were grown on the same farm (36°55′13.12″ N, 114°52′41.52″ E) and under the same agricultural management practices in order to eliminate interference from the other factors.

Prior to the quantification of chemical elements, the sesame seeds were ground into a paste, divided into small aliquots of 250 mg, and then treated by microwave digestion. The digestion treatment was performed in three replicates (*n* = 3). The temperature of the microwave oven was programmed as follows: ramping from 0 °C to 120 °C with a 15 °C/min rate, held for 2 min, then heated to 160 °C with a 8 °C/min rate, maintained for 5 min, ultimately climbed up to 180 °C at 4 °C/min, and maintained for 15 min. After cooling to room temperature, the digested sesame seeds were subjected to multi-element analysis.

For volatile analysis, the sesame seeds were ground into a paste. Approximately 2.5 g of ground sesame seeds was transferred a 20 mL glass vial, followed by the addition of 100 μL 2-methyl-3-heptanone standard solution (0.2 mg/mL in n-hexane). Three independent samples were prepared (*n* = 3). The mixture was vigorously vortexed at 2500 rpm/min for 30 s, and stored until further HS-GC-IMS analysis.

For fatty acid analysis, sesame seeds were prepared following the method developed by Cloos et al. [14], with minor changes. Around 100 mg of sesame seeds was transferred to a 2 mL glass tube, followed by the addition of 1 mL extraction solvent (chloroform/methanol, 2:1), and then ultrasonicated with an ultrasonic bath (KQ-500DE, Kunshan, Jiangsu, China) at a frequency of 50 kHz and temperature of 25 °C for 30 min. Subsequently, the mixed sample solution was centrifugated at 14,000× *g* at 4 °C for 20 min, after which, the resulting supernatant was methylated with 2 mL of 1% sulphuric acid in methanol for 30 min at 80 °C. The FAME fraction was isolated with 1 mL of n-hexane and washed twice with 5 mL of water. A volume of 500 μL extract from each sample was mixed with 25 μL methyl salicylate (internal standard), and then stored at −20 °C for instrumental analysis. Five independent extraction experiments were performed (*n* = 5).

For non-targeted metabolite profiling, sesame seeds were processed following the protocols published by Mi et al. [15] and Benton et al. [16], with minor changes. An aliquot of sesame seeds (60 mg) was processed into paste with liquid nitrogen, followed by the addition of 1 mL of extraction solvent (methanol/acetonitrile/water, 2:2:1), and vigorously vortexed for 30 s. Then, the mixed solution was exposed to ultra-sonification for 30 min, and then incubated under −20 °C for 10 min to precipitate insoluble components. The resulting supernatant was centrifugated at 14,000× *g* at 4 °C for 20 min, and then vacuum dried and re-dissolved in 100 μL of solvent consisting of acetonitrile and water (1:1). The resulting extract solution was vortexed and centrifugated at 14,000× *g* for 15 min at 4 °C. The supernatant was obtained for subsequent instrumental analysis. Five independent extraction experiments were performed (*n* = 5).

### 2.3. ICP-MS Determination of Chemical Elements in the Sesame Seeds

The digested sesame seed samples were subjected to chemical element analysis according to our previously published protocols [15]. An Agilent 7700X ICP-MS instrument (Agilent Technologies, Palo Alto, CA, USA) was utilized with the operation conditions as: spray chamber temperature, 2 °C; forward power, 1280 W; sampling depth: 8 mm; the flow rate of makeup gas, carrier gas, and coolant gas at 1 L/min, 1 L/min, and 1.47 L/min, respectively. The quality control (QC) samples and reagent blank (5% HNO_3_) were inserted within the ICP-MS sequence. They were analyzed under the same working conditions after three tested samples to measure the repeatability and stability of the ICP-MS instrument. All sesame seeds were analyzed in triplicates (*n* = 3).

### 2.4. HS-GC-IMS Analysis of Volatile Flavor Components in the Sesame Seeds

The aroma profiles of the two types of sesame seeds were established by using the FlavourSpec^®^ HS-GC-IMS instrument (G.A.S., Dortmund, Germany). The analytical conditions were set according to those previously published by Hou et al. [17]. The sesame seeds were incubated at 40 °C for 10 min at a rate of 500 r/min. Then, a volume of 1.0 mL was sampled from headspace and injected automatically in a splitless mode using an 85 °C syringe. The aroma components were eluted from an MXT-5 column (15 m × 0.53 mm × 1 μm) at 60 °C along with a carrier gas (N_2_, 99.999% purity). The programmed flow rate was as follows: 0–2 min, 2 mL/min; 2–10 min, increased linearly to 15 mL/min; 10–25 min, increased linearly to 100 mL/min; 25–30 min, 100 mL/min. 

The ionized volatiles were driven to a drift tube with the temperature and voltage at 45 °C and 5 kV, respectively. The volatile constitutes were identified by simultaneously referencing the drift time (DT) and retention index (RI). The RI value of individual analytes was evaluated by referencing the C4-C9 *n*-ketone standards. The DT and RI values of the volatiles were compared to the MS Spectral Database established by NIST version 14.0 (National Institute of Standards and Technology, Washington, DC, USA) and GC-IMS library (Gesellschaft für Analytische Sensorsysteme mbH, Dortmund, Germany). The quantitative analysis of volatiles was conducted based on the comparison of peak area of each volatile with that of the 2-methyl-3-heptanone (0.2 mg/mL). All determinations were conducted in five replicates (*n* = 5).

### 2.5. GC-MS Determination of Fatty Acids in the Sesame Seeds

The GC-MS system (7890A-5975C, Agilent, Santa Clara, CA, USA) was utilized to analyze the individual fatty acid molecules in the sesame seed samples. The FAME components were eluted from a DB-WAX column (Agilent, 30 m × 0.25 mm × 0.25 μm) by using a carrier gas of helium (99.999% purity). The flow rate was set at 1.0 mL/min. The column oven temperature was set in a gradient program: 0–3 min, 50 °C; 3–20 min, 50–220 °C; 20–25 min, 220 °C. The temperatures of inlet, mass spectrometry transfer line, and ion source of mass spectrometric detector (MSD) were maintained at 280, 250, and 230 °C, respectively. The FAME molecules were ionized using a 70-eV electron impact.

MSD ChemStation software was utilized to extract the peak area and retention time of FAMEs. The FAME compounds were annotated by matching the corresponding retention times (RT) with that of the authentic FAME mix standards. The contents of FAMEs were calculated according to the corresponding standard curves and expressed as μg/g dry weight sesame seeds. All sesame seed samples were evaluated in five replicates (*n* = 5).

### 2.6. UHPLC-Q-TOF/MS Profiling of Metabolites in the Sesame Seeds

The composition of metabolites in the investigated sesame seeds was analyzed following our previously described method [15]. An LC instrument (Agilent 1290 Infinity, Santa Clara, CA, USA) equipped with a column (BEH Amide, 100 mm × 2.1 mm × 1.7 μm) was adopted to separate the metabolites. Samples were eluted with a binary mobile phase containing aqueous 25 mM ammonium hydroxide and ammonium acetate solution (A) and acetonitrile (B). The composition of mobile phase was adjusted at 0.3 mL/min following a gradient program: 5% A kept for 0.5 min, increased to 35% A within 6.5 min, then climbed up to 60% A within 1 min and maintained for 1 min, down back to 5% A within 0.1 min, and allowed the column for equilibration for 5 min. The temperature of LC column was set at 25 °C. An aliquot of 2 µL sample was utilized for each run. 

A Triple TOF 6600 mass spectrometer (AB SCIEX, Framingham, MA, USA) was adopted for the detection. The eluted compounds were ionized using an electrospray ionization (ESI) interface under both positive (+) and negative (−) modes. Data were acquired by using both full-scan mode, ranging from *m/z* 600–1000 Da, and information-dependent acquisition (IDA)-triggered product ion scan. The detailed instrumental conditions were set as referenced to the method previously published by our lab [15]. The QC samples were analyzed within the LC-MS sequence to evaluate the stability of the method. All sesame seeds were measured in five replicates (*n* = 5). The identities of metabolites were confirmed by comparing accurate MS (<25 ppm) and tandem MS spectra with those of the database constructed by the lab from Shanghai Applied Protein Technology Co. Ltd (Shanghai, China).

### 2.7. Data Statistics

The Addinsoft XLSTAT-Premium software (v2021, Barcelona, Spain) was utilized in this study for raw data processing and statistics. One-way analysis of variance (ANOVA) was performed to investigate the significance of chemical composition between the two groups of sesame seeds. Data from ANOVA with 95% confidence intervals (*p* < 0.05) were deemed as statistically significant. In addition to univariate statistics, principal component analysis (PCA) was conducted for the evaluation of variations in the concentrations of components between the two groups of sesame seeds. Further, the partial least squares discriminant analysis (PLS-DA) model was established for classification of the two groups of sesame seeds. The variable importance in projection (VIP) score statistically assessed the contribution of individual components to the established PLS-DA model. Components or variables with VIP score > 1 were selected as important contributors for the separation of black and white sesame seed groups. 

## 3. Results and Discussion

### 3.1. Comparison of Element Profiles between Black and White Sesame Seeds

In total, fifty-three chemical elements were quantitatively determined in the sesame seed samples by using an ICP-MS method. The concentration levels and the statistical data of the chemical elements are summarized in Table 1. As shown, calcium (Ca) showed the highest concentration level, followed by phosphorus (P), potassium (K), magnesium (Mg), and iron (Fe). This was partially consistent with previous publications which reported that Ca was present in the sesame seeds at the highest concentration levels, followed in a descending order of K > P > Mg [11,18]. The ANOVA results revealed that there were 16 chemical elements present at statistically varied (*p* < 0.05) contents between the two groups of sesame seeds (Table 1). More specifically, white sesame seeds contained significantly higher levels of B, Na, K, Ca, Cr, Zn, As, Se, Sr, and Mo (10 in total), whereas black sesame seeds contained significantly higher amounts of Mn, Co, Cu, Rb, Cd, and Ba (six in total). These observations were partly varied from those published by Kanu [19], who found that black sesame seeds contained higher levels of Fe, K, Ca, P, Pb, and As than that of white sesame seeds.

**Figure 1:**
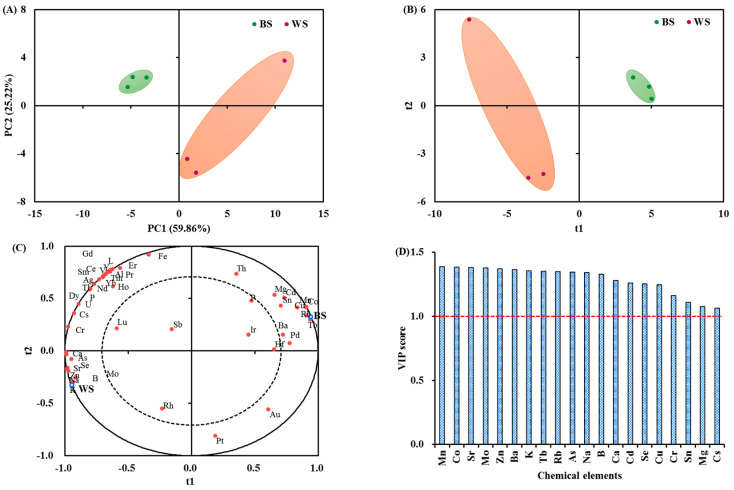
(**A**) Score plot of PCA performed on the chemical elements. (**B**) Score plot of PLS-DA performed on the chemical element data (R^2^X = 0.91, R^2^Y = 1.00 and Q^2^ = 0.99). (**C**) Correlation circle between chemical elements and sesame seeds. (**D**) Bar chart of the VIP scores of the candidate chemical elements for discriminating the two groups of sesame seeds. BS, black sesame seeds; WS, white sesame seeds; VIP, variable importance in projection.

In order to visualize differences between the two types of sesame seeds, multi-variate statistics were carried out relying on the chemical element composition. The PCA results shown in Figure 1A depict a clear separation between the two groups of sesame seeds. The first two principal components (PC) occupied 85.08% of the total variance. A discrimination model of R^2^X = 0.91, R^2^Y = 1.00 and Q^2^ = 0.99 was established to evaluate different chemical elements between the two groups of sesame seeds (Figure 1B). Of these parameters, R^2^ indicates the fitting ability of the established model to the applied data, whereas Q^2^ indicates the reliability of the model to predict a new set of data [15,20]. The correlation between the chemical elements and sesame seeds is illustrated in Figure 1C. It can be seen that chemical elements of Mn (r = 0.99) and Co (r = 0.99) were distributed closely around the black sesame seeds, whereas K (r = 0.99) and Zn (r = 0.99) were closely clustered around white sesame seeds. These observations were consistent with that of the ANOVA analysis results (Table 1). The chemical elements contributing most for the separation of two types of sesame seeds were determined with the cutoff criteria of *p* value < 0.05 and VIP score > 1 [15,20]. As a result, 16 out of 53 investigated chemical elements were screened out: Mn, Co, Sr, Mo, Zn, Ba, K, Rb, As, Na, B, Ca, Cd, Se, Cu, and Cr (Table 1 and Figure 1D).

From the above results, it can be concluded that both black and white sesame seeds are an abundant source of chemical elements, especially for Ca. The concentration levels of 53 chemical elements determined in the sesame seeds of this study varied to different extents from those in the previous publications [3,5,19]. This could be caused by several factors, e.g., different geographic origins and climate conditions, agricultural practices, and genotypes [8]. The sesame seeds analyzed in this study were cultivated under the same agricultural practices and conditions. Therefore, the observed variations could be mainly caused by the seed coat color and genotypes. The black color of sesame seeds is mainly due to the presence of melanin [21]. Plant melanin can chelate many chemical elements, including Cu, Mn, Cd, and Ni [21,22]. Further studies can be performed focusing on the correlation between melanin and the multi-element profiles.

### 3.2. Comparison of Volatile Profiles between Black and White Sesame Seeds

**Figure 2:**
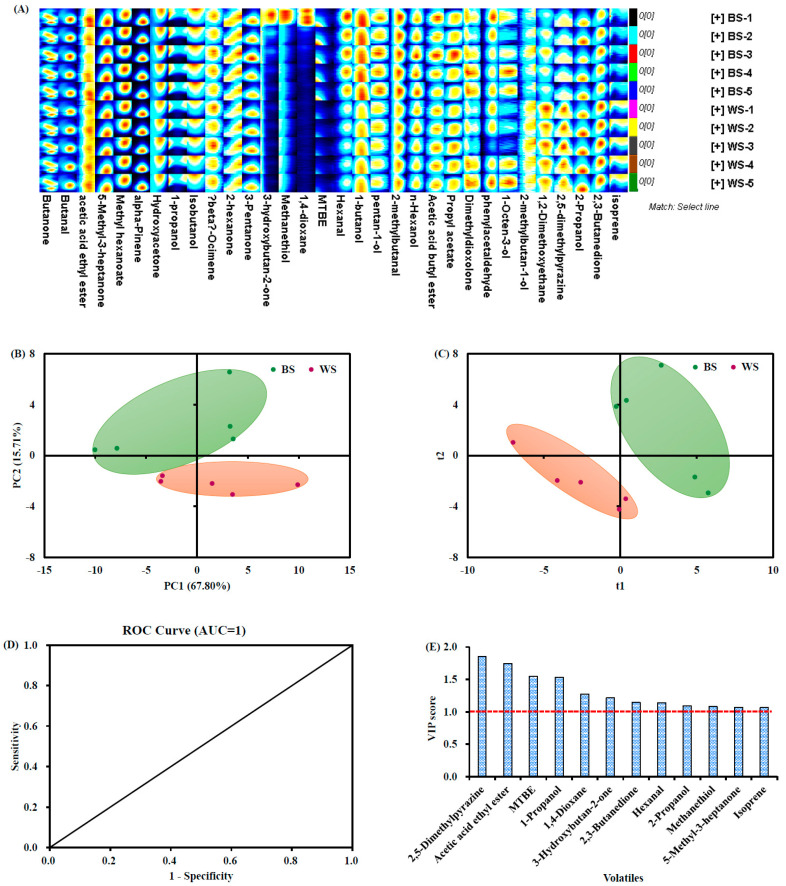
(**A**) Volatile fingerprints of the black and white sesame seeds. The picture was output automatically by the Laboratory Analytical Viewer (LAV) with reporter and three gallery plug-ins. The reporter plug-in enables comparison of the spectral differences, whereas the gallery plot plug-in visually compares the volatile fingerprints between different samples. Each row represents an individual sesame seed sample, whereas each column represents an individual volatile organic compound detected in different samples. The redder color means higher concentration levels of the volatiles. (**B**) Score plot of PCA on the volatile organic compounds. (**C**) Score plot of PLS-DA on the volatile organic compounds (R^2^X = 0.96, R^2^Y = 0.99 and Q^2^ = 0.94). (**D**) ROC curve of the volatile constitutes. (**E**) Bar chart of the VIP scores of the potential aroma markers for separating the two types of sesame seeds. BS, black sesame seeds; WS, white sesame seeds; ROC, receiver operating characteristic; VIP, variable importance in projection.

The volatile flavor components in the sesame seed samples were analyzed by the GC-IMS method. A total of 32 compounds were commonly annotated in the sesame seeds, namely, 9 alcohols, 8 ketones, 5 esters, 4 aldehydes, 2 alkanes, 2 ethers, and 2 other components (Table 2). These data were in good agreement with the GC-MS results published by Cheng et al. [12], who found that alcohols were one of the most abundant flavor families in raw sesame seeds. The presence of alcohol components in sesame seeds contributes to the woody, fruity, alcoholic, balsamic, and green flavors [12,23]. As for the contents of individual volatile compounds, white sesame seeds contained significantly higher levels of acetic acid ethyl ester (37.93 μg/g), 1-propanol (200.48 μg/g), and 2,5-dimethylpyrazine (34.80 μg/g) than that in the black sesame seeds (Table 2 and Figure 2A). No statistical significance (*p* > 0.05) was present in the contents of the other volatile flavor components between the two groups of sesame seeds (Table 2 and Figure 2A). 

Principal components analysis on the volatile data reveals a distinct separation of the two groups of sesame seeds. PC1 and PC2 occupied 83.5% of total variability (see Figure 2B). Figure 2C shows the PLS-DA results with R^2^X = 0.96, R^2^Y = 0.99, and Q^2^ = 0.94, demonstrating that the obtained model was reliable and had sound prediction ability. The receiver operating characteristic (ROC) curve was applied to investigate the sensitivity and (1-specificity) of volatile composition for classification of the two groups of sesame seeds [2]. The area under the receiver operating characteristic curve (AUC) of 1 (Figure 2D) suggests that the volatile compounds have great potential to discriminate the two types of sesame seeds [2]. Finally, three compounds, including acetic acid ethyl ester, 1-propanol, and 2,5-dimethylpyrazine, were identified as the candidate markers being under selection by a combination of both *p* value < 0.05 (Table 2) and VIP score > 1 (Figure 2E). Among them, 2,5-dimethylpyrazine, with barbecue, nut, and scorched flavors, was a main volatile component detected in sesame seeds and derived products [9,23]. Acetic acid ethyl ester, with fresh and fruit flavors, is of great importance to the overall odor of sesame seeds and their products [23]. 

Aroma profile is of great significance for the evaluation of sensory quality of sesame seeds, which would be strongly associated with the overall evaluation of processed products [10,23]. To our knowledge, the HS-GC-IMS method was utilized for the first time to investigate the composition of volatile aroma compounds of sesame seeds. HS-GC-IMS is characterized by minimal sample preparation, intuitive visualization of data, and high sensitivity when compared to the conventional HS-SPME-GC-MS approach [17,24]. This research work not only confirms the effect of genotypes on the flavor quality of sesame seeds, but also reveals the variations in the concentration levels of individual volatile components between the two groups of sesame seeds.

### 3.3. Comparison of Fatty Acid Profiles between Black and White Sesame Seeds

**Figure 3:**
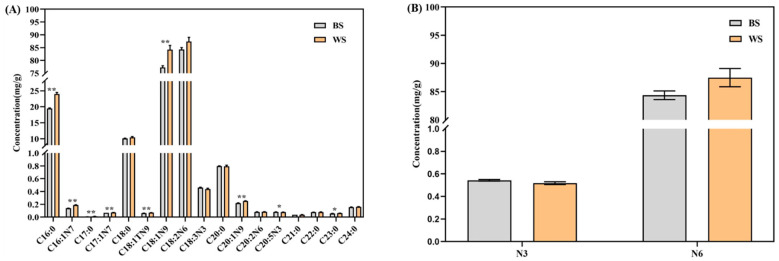

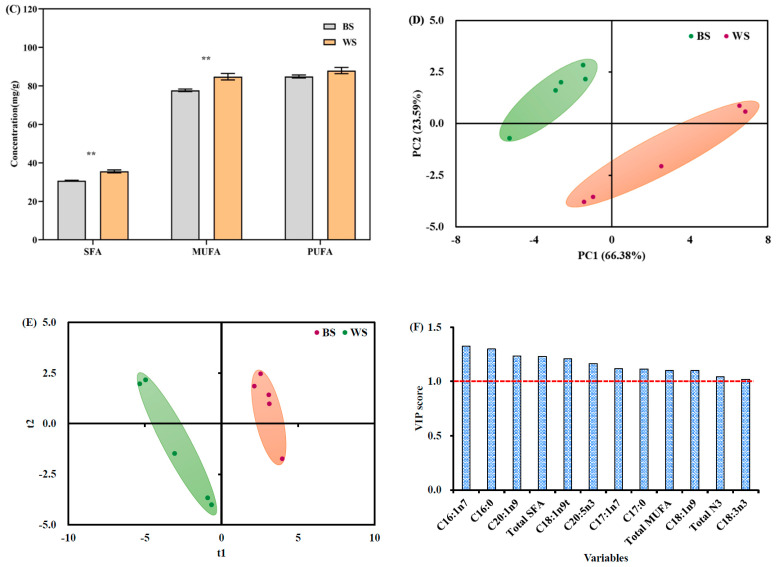
(**A**) Contents of individual FAMEs in the sesame seed samples. (**B**) Contents of n-3 and n-6 FAMEs in the sesame seed samples. (**C**) Contents of different subclasses of FAMEs in the sesame seed samples. (**D**) PCA score plot on the FAME data. (**E**) PLS-DA score plot on the FAME data (R^2^X = 0.90, R^2^Y = 0.99, and Q^2^ = 0.97). (F) Bar chart of the VIP scores of the candidate FAME markers for the two groups of sesame seeds. The level of significance was defined as *p* value < 0.05 * and <0.01 **. BS, black sesame seeds; WS, white sesame seeds; FAME, fatty acid methyl ester; SFA, saturated fatty acids; MUFA, mono-unsaturated fatty acids; PUFA, poly-unsaturated fatty acids; VIP, variable importance in projection.

A total of 40 individual fatty acid methyl esters (FAMEs) covering saturated and mono- and poly-unsaturated FAME species were simultaneously assessed in the sesame seeds. The involved fatty acid compounds and their corresponding calibration curves are summarized in Supplementary Table S1. Among them, C18:2n6 FAME was found to be the most abundant one in the two groups of sesame seeds, followed by C18:1n9, C16:0, and C18:0 FAMEs (Figure 3A). These data generally agree with the previous publications, suggesting that C16:0, C18:0, C18:1n9, and C18:2n6c FAMEs were the dominant constitutes detected in sesame seeds [16,19,25]. Significant variations indicated by *p* value < 0.05 * and <0.01 ** were found in the contents of C16:1n7, C16:0, C20:1n9, C18:1n9t, C17:1n7, C17:0, C18:1n9, C20:5n3, and C23:0 FAMEs (9 in total) between the two groups of sesame seeds (Figure 3A). Except for C20:5n3 FAME, all the other eight FAMEs showed significantly higher levels in white sesame seeds (Figure 3A). The same trend was observed for the contents of total n6 FAMEs (Figure 3B), as well as total saturated, mono-unsaturated, and poly-unsaturated FAMEs (Figure 3C). All these variations can plausibly be ascribed by the varied sesame seed genotypes [8,26]. 

PCA was also applied on the fatty acid data to evaluate the group diversity of sesame seeds [27,28]. The PC1 (66.38%) and PC2 (23.59%) score plot for all sesame seed samples is shown in Figure 3D. There was a clear distinction between the two groups of sesame seeds. Obvious differences were also found in the score plot of PLS-DA on the fatty acid composition of sesame seeds, as shown in Figure 3E. The VIP values of the detected FAMEs were generated from the constructed PLS-DA model. In total, nine individual FAMEs, and total saturated, mono-unsaturated, and n3 FAMEs had VIP value > 1 (Figure 3F). Taking *p* value < 0.05 into consideration, C16:1n7, C16:0, C20:1n9, C18:1n9t, C17:1n7, C17:0, C18:1n9, C20:5n3, and total saturated and mono-unsaturated FAMEs were finally screened out as important indicators for differentiating the two groups of sesame seeds (Figure 3A,C,F). 

Taken together, our data clearly demonstrate that differences were present between the two types of sesame seeds in the contents of FAME species. It is noteworthy that although most FAMEs exhibited higher levels in white sesame seeds, n-3 poly-unsaturated FAMEs, including C18:3n3 and C20:5n3, were more abundant in black sesame seeds. Several reports have found that n-3 long chain poly-unsaturated fatty acids (LC-PUFA) are involved in many important biological activities, for example, anti-inflammation [29], anti-cancer [30], anti-aging [31], and lowering the risk of cardiovascular diseases [32]. It was hypothesized that the expression levels of genes associated with LC-PUFA synthesis could be varied between the two groups of sesame seeds. Further studies are still needed to explore the underlying mechanisms behind these observations. 

### 3.4. Comparison of Metabolite Profiles between Black and White Sesame Seeds

**Figure 4:**
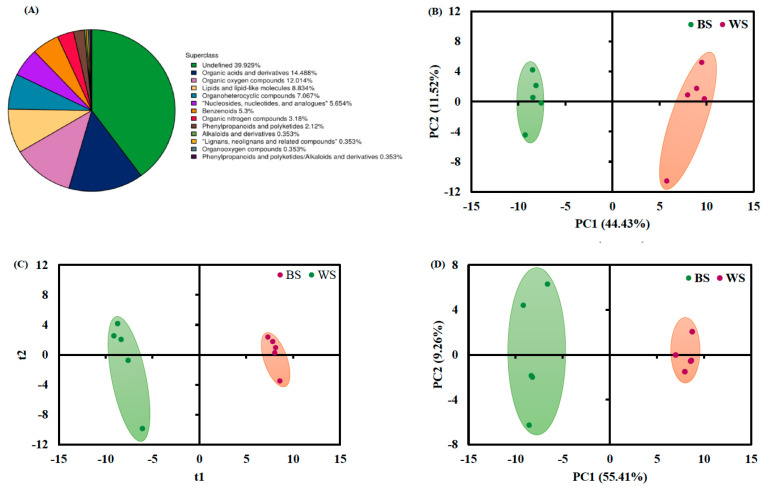

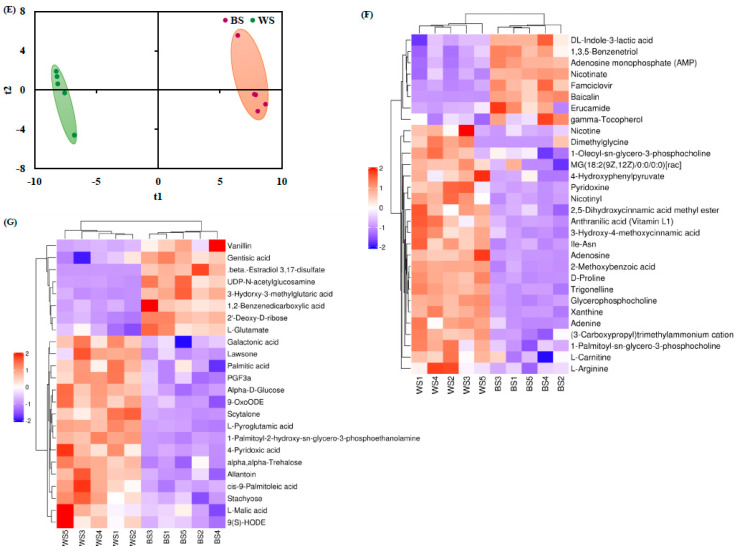
(**A**) Classification of metabolites identified in black and white sesames. (**B**) PCA score plot on the metabolites identified under ESI (+) mode. (**C**) PLS-DA score plot performed on the metabolites identified under ESI (+) mode (R^2^X = 0.55, R^2^Y = 0.99 and Q^2^ = 0.98). (**D**) PCA score plot performed on the metabolites identified under ESI (−) mode. (**E**) PLS-DA score plot performed on the metabolites identified under ESI (−) mode (R^2^X = 0.62, R^2^Y = 0.99, and Q^2^ = 0.98). (**F**) Heatmap of significantly different metabolites detected under ESI (+) mode between the two types of sesame seeds. (**G**) Heatmap of significantly varied metabolites identified under ESI (−) mode between the two types of sesame seeds. The differential metabolites were screened out, relying on the cutoff criteria of adjusted *p* value < 0.05 and VIP score > 1. Red color demonstrates significantly upregulated levels of metabolites, whereas the blue color demonstrates significantly downregulated levels of metabolites. BS, black sesame seeds; WS, white sesame seeds; VIP, variable importance in projection.

A total of 161 and 122 compounds were annotated in the positive (+) and negative (−) ESI mass spectrometry, respectively. The defined compounds (60.07%) were categorized into 12 super classes, as illustrated in Figure 4A with different colors. The largest proportion of 14.49% was occupied by organic acids and derived compounds, followed by organic oxygen molecules at 12.01%, and lipids and derived compounds at 8.83% (Figure 4A). These observations are generally consistent with the previous report, which found that lipids and derived compounds, organic acids, and organo-heterocyclic molecules were the dominant groups present in the sesame seed samples [4]. 

The score plots obtained from multivariate statistical analyses on the metabolite data of sesame seed samples are shown in Figure 4B–E. A clear separation between the two groups of sesame seeds was observed from these score plots. A total of 54 statistically-altered metabolite compounds were selected according to the criteria of *p* value < 0.05 from univariate analysis and VIP score > 1 [15]. Among them, 38 differential metabolites showed significantly higher abundance levels in white sesame seeds than those in black sesame seeds (Figure 4F positive and Figure 4G negative ion modes). Furthermore, the fold change (FC) of abundance was taken into consideration. The metabolites with FC either > 2 or < 0.5, adjusted *p* value < 0.05, and VIP score > 1 are summarized in Supplementary Table S2. As shown, the most abundant molecules in black sesame seeds were β-estradiol 3,17-disulfate (FC = 305.57), and baicalin (FC = 29.69) (Supplementary Table S2). Baicalin, a flavonoid Kampo compound, has been widely studied due to its important biological functions, including anti-inflammatory, antiviral, anti-tumor, and photoprotective effects [33]. As for white sesame seeds, the top two up-regulated compounds were dimethylglycine and trigonelline (Supplementary Table S2). Both dimethylglycine and trigonelline have been reported to be associated with therapeutic potential for many diseases, especially for diabetes [34]. 

As a summary, these findings demonstrate that the two groups of sesame seeds were different from each other in terms of metabolite composition and abundance. Other researchers also observed the metabolite variations among different groups of sesame seeds, and reasoned that these differences could possibly be caused by the genetic (e.g., CHS, F3H, DFR, and MYB) and environmental influences, and mostly their interactions [2,4,8]. These two kinds of sesame seeds were characterized by metabolites with different biological potentials. Our results thus merit further investigation and comparison on the biological activities of the two groups of sesame seeds.

## 4. Conclusions

The current research achieves a relatively comprehensive chemical fingerprint, comprising 53 chemical elements, 32 volatile aroma compounds, 40 individual fatty acids, and 283 metabolites of sesame seeds. As compared to black sesame seeds, the white group generally contained more chemical elements, volatile flavor compounds, fatty acids, and metabolites. An interesting observation is that n-3 long chain poly-unsaturated fatty acids were more abundant in the black group than in the white one. We confirmed significant differences and identified potential markers for the discrimination of black and white sesame seeds. These findings could be of significance for the development of novel products, and also provide a rationale for the price and quality variation between the two groups of sesame seeds.

## Figures and Tables

**Table 1:** Contents and statistical results of chemical elements determined in the sesame seeds.

Chemical Elements	Black Sesame Seeds	White Sesame Seeds	*p* Value
B	8.00 ± 0.22 ^b^	9.23 ± 0.22 ^a^	<0.01
Na	15.01 ± 0.84 ^b^	35.72 ± 3.66 ^a^	<0.01
Mg	3266.97 ± 37.89 ^a^	3143.82 ± 84.45 ^a^	0.08
Al	28,570.61 ± 1994.83 ^a^	40,979.52 ± 23196.22 ^a^	0.41
P	6564.94 ± 99.60 ^a^	6415.67 ± 163.69 ^a^	0.25
K	5186.90 ± 51.18 ^b^	5721.57 ± 63.21 ^a^	<0.01
Ca	9758.70 ± 326.90 ^b^	11,692.50 ± 538.44 ^a^	0.01
V	89.91 ± 8.24 ^a^	114.97 ± 56.13 ^a^	0.49
Cr	80.02 ± 9.60 ^b^	186.45 ± 57.98 ^a^	0.03
Mn	18,358.41 ± 189.85 ^a^	9936.66 ± 712.65 ^b^	<0.01
Fe	71.14 ± 4.34 ^a^	83.42 ± 34.94 ^a^	0.58
Co	195.67 ± 0.99 ^a^	66.60 ± 11.43 ^b^	<0.01
Ni	772.25 ± 656.80	N.A.	N.A.
Cu	18,523.51 ± 281.40 ^a^	17,638.38 ± 255.02 ^b^	0.02
Zn	41,249.64 ± 670.53 ^b^	49,721.01 ± 780.07 ^a^	<0.01
As	14.42 ± 5.78 ^b^	93.20 ± 12.30 ^a^	<0.01
Se	111.60 ± 6.39 ^b^	135.36 ± 6.33 ^a^	0.01
Rb	8172.23 ± 686.90 ^a^	4799.52 ± 49.31 ^b^	<0.01
Sr	41,527.71 ± 1350.69 ^b^	78,744.99 ± 791.66 ^a^	<0.01
Y	21.08 ± 11.02 ^a^	45.15 ± 52.30 ^a^	0.48
Mo	590.68 ± 33.16 ^b^	1636.58 ± 40.24 ^a^	<0.01
Ru	N.A.	N.A.	N.A.
Rh	N.A.	3.13 ± 5.42	N.A.
Pd	16.72 ± 4.45 ^a^	11.12 ± 1.21 ^a^	0.10
Ag	3.61 ± 0.59 ^a^	4.26 ± 1.11 ^a^	0.42
Cd	76.49 ± 7.50 ^a^	58.94 ± 3.25 ^b^	0.02
Sn	488.73 ± 194.94 ^a^	161.18 ± 101.29 ^a^	0.06
Sb	2.02 ± 0.24 ^a^	2.14 ± 0.81 ^a^	0.81
Te	N.A.	N.A.	N.A.
Cs	7.67 ± 0.22 ^a^	19.87 ± 9.00 ^a^	0.08
Ba	32,457.92 ± 2355.45 ^a^	15,442.80 ± 313.27 ^b^	<0.01
La	27.04 ± 7.86 ^a^	54.08 ± 61.51 ^a^	0.49
Ce	36.17 ± 10.12 ^a^	63.60 ± 44.69 ^a^	0.36
Pr	5.13 ± 2.02 ^a^	10.63 ± 11.29 ^a^	0.45
Nd	17.66 ± 8.87 ^a^	42.10 ± 44.45 ^a^	0.40
Sm	3.42 ± 1.21 ^a^	9.03 ± 10.10 ^a^	0.39
Eu	2.33 ± 0.47 ^a^	2.34 ± 1.60 ^a^	0.99
Gd	3.57 ± 1.20 ^a^	9.45 ± 11.44 ^a^	0.43
Tb	82.07 ± 14.25	N.A.	N.A.
Dy	2.78 ± 0.93 ^a^	6.79 ± 7.93 ^a^	0.43
Ho	0.69 ± 0.28 ^a^	1.36 ± 1.59 ^a^	0.52
Er	1.51 ± 0.47 ^a^	3.40 ± 4.51 ^a^	0.51
Tm	0.26 ± 0.10 ^a^	0.58 ± 0.54 ^a^	0.38
Yb	1.21 ± 0.49 ^a^	3.86 ± 3.75 ^a^	0.29
Lu	149.29 ± 131.00 ^a^	249.43 ± 118.28 ^a^	0.38
Hf	23.48 ± 10.05 ^a^	10.05 ± 11.02 ^a^	0.19
Ir	1.86 ± 3.22	N.A.	N.A.
Pt	0.94 ± 0.11 ^a^	0.97 ± 0.16 ^a^	0.82
Au	10.37 ± 0.54 ^a^	9.72 ± 1.23 ^a^	0.45
Tl	0.61 ± 0.09 ^a^	0.95 ± 0.42 ^a^	0.25
Pb	48.73 ± 3.86 ^a^	68.72 ± 25.09 ^a^	0.24
Th	21.10 ± 6.09 ^a^	13.66 ± 8.01 ^a^	0.27
U	1.38 ± 0.13 ^a^	5.89 ± 3.99 ^a^	0.12

Note: All values are expressed as the mean (*n* = 3) ± standard deviation. Different letters in the same row represent significant difference at *p* < 0.05. The levels of B, Na, Mg, P, K, Ca, and Fe are expressed in microgram per gram (μg/g) of sesame seeds, whereas the other elements are given in microgram per kilogram (μg/kg) of sesame seeds. N.A., not available.

**Table 2:** Contents and statistical results of the volatile compounds determined in the black and white sesame seeds.

No.	Category	Compounds	CAS#	Formula	MW	RI	RT (min)	DT (a.u.)	Contents (μg/g)		*p* Value
									BS	WS	
1	Aldehydes	Phenylacetaldehyde	C122781	C_8_H_8_O	120.2	1014.3	8.77	1.27	52.91 ± 10.60 ^a^	60.62 ± 27.22 ^a^	0.57
2		Hexanal	C66251	C_6_H_12_O	100.2	792.8	4.07	1.26	62.63 ± 14.69 ^a^	55.63 ± 9.52 ^a^	0.40
3		2-Methylbutanal	C96173	C_5_H_10_O	86.1	661.8	2.71	1.15	137.44 ± 32.61 ^a^	134.68 ± 24.12 ^a^	0.56
4		Butanal	C123728	C_4_H_8_O	72.1	619.5	2.43	1.28	359.35 ± 83.83 ^a^	416.43 ± 75.68 ^a^	0.29
5	Ketones	Dimethyldioxolone	C37830903	C_5_H_6_O_3_	114.1	966.4	7.31	1.17	45.75 ± 14.58 ^a^	47.11 ± 15.21 ^a^	0.89
6		5-Methyl-3-heptanone	C541855	C_8_H_16_O	128.2	945	6.80	1.28	126.53 ± 56.71 ^a^	98.99 ± 26.43 ^a^	0.35
7		2-Hexanone	C591786	C_6_H_12_O	100.2	785.7	3.96	1.18	40.41 ± 9.22 ^a^	42.62 ± 8.11 ^a^	0.70
8		3-Hydroxybutan-2-one	C513860	C_4_H_8_O_2_	88.1	716.6	3.19	1.06	73.77 ± 74.24 ^a^	27.56 ± 21.89 ^a^	0.22
9		Hydroxyacetone	C116096	C_3_H_6_O_2_	74.1	626.4	2.47	1.22	554.19 ± 143.03 ^a^	617.66 ± 117.44 ^a^	0.47
10		3-Pentanone	C96220	C_5_H_10_O	86.1	651.7	2.64	1.35	100.92 ± 27.05 ^a^	106.51 ± 18.62 ^a^	0.71
11		2,3-Butanedione	C431038	C_4_H_6_O_2_	86.1	585.4	2.20	1.19	608.86 ± 145.45 ^a^	802.22 ± 125.24 ^a^	0.05
12		Butanone	C78933	C_4_H_8_O	72.1	548.6	1.96	1.04	1352.07 ± 314.15 ^a^	1605.94 ± 267.51 ^a^	0.21
13	Alcohols	3-Octenol	C3391864	C_8_H_16_O	128.2	965.2	7.28	1.73	27.98 ± 10.65 ^a^	30.00 ± 12.85 ^a^	0.79
14		*n*-Hexanol	C111273	C_6_H_14_O	102.2	880.1	5.37	1.32	66.88 ± 16.65 ^a^	76.06 ± 10.57 ^a^	0.33
15		2-Methylbutan-1-ol	C137326	C_5_H_12_O	88.1	766.3	3.74	1.23	14287.18 ± 5954.86 ^a^	18509.37 ± 6883.96 ^a^	0.33
16		1-Pentanol	C71410	C_5_H_12_O	88.1	768.2	3.76	1.25	31.01 ± 7.69 ^a^	32.94 ± 5.90 ^a^	0.67
17		1-Butanol	C71363	C_4_H_10_O	74.1	610.3	2.36	1.38	9.59 ± 5.20 ^a^	8.77 ± 3.41 ^a^	0.78
18		Isobutanol	C78831	C_4_H_10_O	74.1	591.9	2.24	1.38	222.29 ± 48.70 ^a^	254.58 ± 45.73 ^a^	0.31
19		2-Propanol	C67630	C_3_H_8_O	60.1	534.8	1.86	1.09	303.53 ± 59.56 ^a^	354.45 ± 49.15 ^a^	0.18
20		1-Propanol	C71238	C_3_H_8_O	60.1	521.4	1.78	1.13	118.34 ± 27.38 ^b^	200.48 ± 34.55 ^a^	0.003
21		Methanethiol	C74931	CH_4_S	48.1	468.4	1.43	1.04	51.50 ± 50.90 ^a^	40.69 ± 10.19 ^a^	0.65
22	Esters	Methyl hexanoate	C106707	C_7_H_14_O_2_	130.2	929	6.42	1.28	441.05 ± 95.94 ^a^	541.71 ± 94.33 ^a^	0.13
23		Acetic acid butyl ester	C123864	C_6_H_12_O_2_	116.2	806.3	4.27	1.23	28.07 ± 7.77 ^a^	27.96 ± 4.60 ^a^	0.98
24		Propyl acetate	C109604	C_5_H_10_O_2_	102.1	657.4	2.68	1.48	116.08 ± 33.10 ^a^	127.96 ± 25.29 ^a^	0.54
25		1,2-Dimethoxyethane	C110714	C_4_H_10_O_2_	90.1	638.8	2.55	1.10	40.55 ± 11.53 ^a^	45.91 ± 12.16 ^a^	0.50
26		Acetic acid ethyl ester	C141786	C_4_H_8_O_2_	88.1	609.4	2.36	1.34	10.56 ± 4.31 ^b^	37.93 ± 8.80 ^a^	<0.01
27	Alkanes	α-Pinene	C80568	C_10_H_16_	136.2	936.9	6.61	1.69	1710.80 ± 390.53 ^a^	2081.38 ± 359.40 ^a^	0.16
28		Isoprene	C78795	C_5_H_8_	68.1	528.8	1.82	1.21	68.54 ± 26.98 ^a^	82.22 ± 13.58 ^a^	0.34
29	Ethers	1,2-Dimethoxyethane	C110714	C_4_H_10_O_2_	90.1	638.8	2.55	1.10	40.55 ± 11.53 ^a^	45.91 ± 12.16 ^a^	0.50
30		MTBE	C1634044	C_5_H_12_O	88.1	566.5	2.07	1.35	37.32 ± 8.12 ^a^	42.57 ± 8.38 ^a^	0.34
31	Others	2,5-Dimethylpyrazine	C123320	C_6_H_8_N_2_	108.1	940.3	6.69	1.50	9.93 ± 2.14 ^b^	34.80 ± 5.87 ^a^	<0.01
32		1,4-Dioxane	C123911	C_4_H_8_O_2_	88.1	710.6	3.13	1.33	29.92 ± 33.15 ^a^	5.59 ± 2.40 ^a^	0.14

Note: All values are expressed as the mean (*n* = 5) ± standard deviation. Different letters in the same row represent significant difference at *p* < 0.05. MW, molecular weight; RI, retention index; RT, retention time; DT, drift time; BS, black sesame seeds; WS, white sesame seeds.

## Data Availability

The authors confirm that the data supporting the findings of this study are available within the article and its Supplementary Materials.

## Supplementary Materials

The following supporting information can be downloaded at: https://www.mdpi.com/article/10.3390/foods11142042/s1, Table S1: Information of individual fatty acid methyl esters quantified in the black and white sesame seeds; Table S2: Summary of differential metabolites between black and white sesame seeds.

## References

[1] Botelho J.R.S., Medeiros N.G., Rodrigues A.M.C., Araújo M.E., Machado N.T., Santos A.G., Santos I.R., Gomes-Leal W., Carvalho R.N. (2014). Black sesame (*Sesamum indicum* L.) seeds extracts by CO_2_ supercritical fluid extraction: Isotherms of global yield, kinetics data, total fatty acids, phytosterols and neuroprotective effects. J. Supercrit. Fluids.

[2] Kim S.-Y., Kim E., Shin B.K., Seo J.-A., Kim Y.-S., Lee D.Y., Choi H.-K. (2020). NMR-based metabolic profiling discriminates the geographical origin of raw sesame seeds. Food Control.

[3] Yadav A., Saini I., Kaushik P., Ansari M.A., Khan M.R., Haq N. (2021). Effects of arbuscular mycorrhizal fungi and P-solubilizing Pseudomonas fluorescence (ATCC-17400) on morphological traits and mineral content of sesame. Saudi J. Biol. Sci..

[4] Lee B.M., Lee E.M., Kang D.J., Seo J.-A., Choi H.-K., Kim Y.-S., Lee D.Y. (2020). Discovery study of integrative metabolic profiles of sesame seeds cultivated in different countries. LWT-Food Sci. Technol..

[5] Dravie E.E., Kortei N.K., Essuman E.K., Tettey C.O., Boakye A.A., Hunkpe G. (2020). Antioxidant, phytochemical and physicochemical properties of sesame seed (*Sesamum indicum* L). Sci. Afr..

[6] Parsaeian M., Shahabi M., Hassanpour H. (2020). The integration of image processing and artificial neural network to estimate four fatty acid contents of sesame oil. LWT.

[7] De Magalhães B.E.A., Santana D.D.A., Silva I.M.D.J., Minho L.A.C., Gomes M., Almeida J.R.G.D.S., dos Santos W.N.L. (2020). Determination of phenolic composition of oilseed whole flours by HPLC-DAD with evaluation using chemometric analyses. Microchem. J..

[8] Morris J.B., Wang M.L., Tonnis B.D. (2021). Variability for oil, protein, lignan, tocopherol, and fatty acid concentrations in eight sesame (*Sesamum indicum* L.) genotypes. Ind. Crop. Prod..

[9] Shen Y., Hu L.-T., Xia B., Ni Z.-J., Elam E., Thakur K., Zhang J.-G., Wei Z.-J. (2021). Effects of different sulfur-containing substances on the structural and flavor properties of defatted sesame seed meal derived Maillard reaction products. Food Chem..

[10] Wu Z., Qin D., Duan J., Li H., Sun J., Huang M., Sun B. (2021). Characterization of benzenemethanethiol in sesame-flavour baijiu by high-performance liquid chromatography-mass spectrometry and sensory science. Food Chem..

[11] Wacal C., Ogata N., Sasagawa D., Handa T., Basalirwa D., Acidri R., Ishigaki T., Yamamoto S., Nishihara E. (2019). Seed yield, crude protein and mineral nutrient contents of sesame during a two-year continuous cropping on upland field converted from a paddy. Field Crop. Res..

[12] Cheng R., Liao X., Addou A.M., Qian J., Wang S., Cheng Z., Wang L., Huang J. (2021). Effects of “nine steaming nine sun-drying” on proximate composition, oil properties and volatile compounds of black sesame seeds. Food Chem..

[13] Chun Y.S., Kim S.-Y., Kim M., Lim J.Y., Shin B.K., Kim Y.-S., Lee D.Y., Seo J.-A., Choi H.-K. (2021). Mycobiome analysis for distinguishing the geographical origins of sesame seeds. Food Res. Int..

[14] Cloos A.-S., Ghodsi M., Stommen A., Vanderroost J., Dauguet N., Pollet H., D’Auria L., Mignolet E., Larondelle Y., Terrasi R. (2020). Interplay between plasma membrane lipid alteration, oxidative stress and calcium-based mechanism for extracellular vesicle biogenesis from erythrocytes during blood storage. Front. Physiol..

[15] Mi S., Yu W., Li J., Liu M., Sang Y., Wang X. (2020). Characterization and discrimination of chilli peppers based on multi-element and non-targeted metabolomics analysis. LWT.

[16] Benton H.P., Ivanisevic J., Mahieu N.G., Kurczy M.E., Johnson C.H., Franco L., Rinehart D., Valentine E., Gowda H., Ubhi B.K. (2014). Autonomous metabolomics for rapid metabolite identification in global profiling. Anal. Chem..

[17] Hou H., Liu C., Lu X., Fang D., Hu Q., Zhang Y., Zhao L. (2021). Characterization of flavor frame in shiitake mushrooms (*Lentinula edodes*) detected by HS-GC-IMS coupled with electronic tongue and sensory analysis: Influence of drying techniques. LWT-Food Sci. Technol..

[18] Tenyang N., Ponka R., Tiencheu B., Djikeng F.T., Azmeera T., Karuna M.S., Prasad R.B., Womeni H.M. (2017). Effects of boiling and roasting on proximate composition, lipid oxidation, fatty acid profile and mineral content of two sesame varieties commercialized and consumed in Far-North Region of Cameroon. Food Chem..

[19] Kanu P.J. (2011). Biochemical analysis of black and white sesame seeds from China. Am. J. Biochem. Mol. Biol..

[20] Mi S., Shang K., Li X., Zhang C.H., Liu J.Q., Huang D.Q. (2019). Characterization and discrimination of selected China’s domestic pork using an LC-MS-based lipidomics approach. Food Control.

[21] Fei X., Qi Y., Lei Y., Wang S., Hu H., Wei A. (2021). Transcriptome and metabolite analysis reveals key genes for melanin synthesis during the development of *Zanthoxylum bungeanum* seeds. Ind. Crop. Prod..

[22] Bai L., Cheng X., Xu J., Wang X., Zhao H., Tao Y., Huang H. (2019). Black sesame pigment extract from sesame dregs by subcritical CO_2_: Extraction optimization, composition analysis, binding copper and antioxidant protection. LWT-Food Sci. Technol..

[23] Li C., Hou L. (2018). Review on volatile flavor components of roasted oilseeds and their products. Grain Oil Sci. Technol..

[24] Chen X., Chen H., Xiao J., Liu J., Tang N., Zhou A. (2020). Variations of volatile flavour compounds in finger citron (*Citrus medica* L. var. sarcodactylis) pickling process revealed by E-nose, HS-SPME-GC-MS and HS-GC-IMS. Food Res. Int..

[25] Dar A.A., Kancharla P.K., Chandra K., Sodhi Y.S., Arumugam N. (2019). Assessment of variability in lignan and fatty acid content in the germplasm of *Sesamum indicum* L.. J. Food Sci. Technol..

[26] Ahmed I.A.M., Uslu N., Özcan M.M., Al Juhaimi F., Ghafoor K., Babiker E.E., Osman M.A., Alqah H.A. (2021). Effect of conventional oven roasting treatment on the physicochemical quality attributes of sesame seeds obtained from different locations. Food Chem..

[27] Akin G., Elmas N.K., Arslan F.N., Yılmaz I., Kenar A. (2019). Chemometric classification and quantification of cold pressed grape seed oil in blends with refined soybean oils using attenuated total reflectance–mid infrared (ATR–MIR) spectroscopy. LWT-Food Sci. Technol..

[28] Xing C., Yuan X., Wu X., Shao X., Yuan J., Yan W. (2019). Chemometric classification and quantification of sesame oil adulterated with other vegetable oils based on fatty acids composition by gas chromatography. LWT-Food Sci. Technol..

[29] Salsinha A.S., Rodríguez-Alcalá L.M., Relvas J.B., Pintado M.E. (2021). Fatty acids role on obesity induced hypothalamus inflammation: From problem to solution—A review. Trends Food Sci. Technol..

[30] Pfister E., Smith R., Lane M.A. (2021). N-3 Polyunsaturated fatty acid ethyl esters decrease the invasion, but not the proliferation, of human colorectal cancer cells via a PI3K-dependent mechanism in vitro. Prostaglandins Leukot. Essent. Fat. Acids.

[31] Ma W.-J., Li H., Zhang W., Zhai J., Li J., Liu H., Guo X.-F., Li D. (2021). Effect of n-3 polyunsaturated fatty acid supplementation on muscle mass and function with aging: A meta-analysis of randomized controlled trials. Prostaglandins Leukot. Essent. Fat. Acids.

[32] Mallick R., Basak S., Duttaroy A.K. (2021). Fatty acids and evolving roles of their proteins in neurological, cardiovascular disorders and cancers. Prog. Lipid Res..

[33] Gong W.-Y., Zhao Z.-X., Liu B.-J., Lu L.-W., Dong J.-C. (2017). Exploring the chemopreventive properties and perspectives of baicalin and its aglycone baicalein in solid tumors. Eur. J. Med. Chem..

[34] Zhou J., Chan L., Zhou S. (2012). Trigonelline: A plant alkaloid with therapeutic potential for diabetes and central nervous system disease. Curr. Med. Chem..

