# Supplementary material for: Discrimination of Black and White Sesame Seeds Based on Targeted and Non-Targeted Platforms with Chemometrics: From Profiling towards Identification of Chemical Markers
Source: Foods. 2022 Jul 11;11(14):2042. doi: 10.3390/foods11142042 (PMC9315883; doi:10.3390/foods11142042)
Supplement: Supplementary file 1 [file foods-11-02042-s001.zip › foods-1793872-supplementary.pdf]

Supplementary Table S1

Information of individual fatty acid methyl esters quantified in the black and white sesame seeds.

No.	Fatty acid methyl esters	Symbols	Calibration curves	R^2 value	Linear dynamic range (μg/mL)
1	Methyl butyrate	C4:0	y = 0.68x + 0.01	0.9996	0.002 - 50
2	Methyl hexanoate	C6:0	y = 2.15x + 2.40E-004	0.9997	0.002 - 50
3	Methyl octanoate	C8:0	y = 2.81x + 3.55E-004	0.9997	0.002 - 50
4	Methyl decanoate	C10:0	y = 2.97x + 4.41E-004	0.9997	0.004 - 100
5	Methyl undecanoate	C11:0	y = 2.94x + 3.13E-004	0.9998	0.002 - 50
6	Methyl dodecanoate	C12:0	y = 2.92x + 3.92E-004	0.9998	0.004 - 100
7	Methyl tridecanoate	C13:0	y = 2.77x + 3.06E-004	0.9997	0.002 - 50
8	Methyl myristate	C14:0	y = 2.58x + 0.015441	0.9996	0.002 - 50
9	Methyl myristoleate	C14:1n5	y = 1.09x - 6.42E-004	0.9993	0.002 - 50
10	Methyl pentadecanoate	C15:0	y = 2.53x + 1.38E-004	0.9997	0.002 - 50
11	Methyl cis-10-pentadecenoate	C15:1n5	y = 0.98x + 1.15E-004	0.9998	0.002 - 50

12	Methyl palmitate	C16:0	y = 2.54x + 0.07	0.9997	0.004 - 100
13	Methyl palmitoleate	C16:1n7	y = 0.77x + 0.01	0.9997	0.002 - 50
14	Methyl heptadecanoate	C17:0	y = 2.40x - 8.81E-004	0.9997	0.004 - 100
15	Methyl cis-10-heptadecenoate	C17:1n7	y = 0.75x + 1.37E-004	0.9997	0.002 - 50
16	Methyl stearate	C18:0	y = 2.44x + 0.08	0.9996	0.004 - 100
17	Methyl elaidate	C18:1n9t	y = 0.78x - 0.004	0.9996	0.002 - 50
18	Methyl oleate	C18:1n9	y = 0.72x + 0.10	0.9995	0.004 - 100
19	Methyl linolelaidate	C18:2n6t	y = 0.96x - 0.02	0.9996	0.002 - 50
20	Methyl linoleate	C18:2n6	y = 0.96x - 0.03	0.9996	0.002 - 50
21	Methyl γ-linolenate	C18:3n6	y = 0.78x + 3.12E-004	0.9999	0.004 - 100
22	Methyl linolenate	C18:3n3	y = 1.05x - 0.004	0.9998	0.002 - 50
23	Methyl arachidate	C20:0	y = 1.87x + 1.53E-004	0.9998	0.004 - 100
24	cis-11-Eicosenoic acid methyl ester	C20:1n9	y = 0.83x - 0.01	0.9999	0.002 - 50
25	cis-11,14-Eicosadienoic acid methyl ester	C20:2n6	y = 0.91x - 0.02	0.9995	0.002 - 50

26	Methyl heneicosanoate	C21:0	y = 1.55x + 0.001	0.9999	0.002 - 50
27	cis-8,11,14-Eicosatrienoic acid methyl ester	C20:3n6	y = 0.88x - 0.03	0.9995	0.002 - 50
28	Methyl arachidonate	C20:4n6	y = 0.95x - 0.05	0.9995	0.002 - 50
29	cis-11,14,17-Eicosatrienoic acid methyl ester	C20:3n3	y = 1.12x - 0.06	0.9995	0.002 - 50
30	Methyl behenate	C22:0	y = 1.60x - 2.48E-004	0.9995	0.004 - 100
31	cis-5,8,11,14,17-Eicosapentaenoic acid methyl ester	C20:5n3	y = 1.08x - 0.01	0.9999	0.002 - 50
32	Methyl erucate	C22:1n9	y = 0.94x - 0.01	0.9999	0.002 - 50
33	cis-13,16-Docosadienoic acid methyl ester	C22:2n6	y = 0.55x + 3.97E-005	0.9994	0.002 - 50
34	Methyl tricosanoate	C23:0	y = 1.88x - 0.01	0.9999	0.002 - 50
35	Methyl docosatetraenoate	C22:4n6	y = 1.01x - 0.03	0.9992	0.002 - 50
36	Methyl docosapentaenoate	C22:5n6	y = 1.02x - 0.03	0.9992	0.002 - 50
37	Methyl tetracosanoate	C24:0	y = 1.79x - 0.02	0.9998	0.004 - 100
38	Methyl docosapentaenoate	C22:5n3	y = 1.03x - 0.04	0.9999	0.002 - 50
39	Methyl cis-15-tetracosenoate	C24:1n9	y = 1.04x - 0.05	0.9997	0.002 - 50

40	cis-4,7,10,13,16,19-Docosahexaenoic acid methyl ester	C22:6n3	y = 1.10x - 0.07	0.9995	0.002 - 50

Supplementary Table S2

Summary of differential metabolites between black and white sesame seeds.

No.	Compounds	Adduct ions	*m/z*	RT (min)	Fold change (BS/WS)	*p* value	VIP
1	Glycerophosphocholine	M^+	258.1103	7.08	0.41	< 0.01	4.41
2	Trigonelline	M^+	138.0539	4.92	0.19	< 0.01	10.00
3	D-Proline	$[M+H]^+$	116.0694	5.20	0.39	< 0.01	2.41
4	Baicalin	$[M+H-H_2O]^+$	411.0689	0.50	29.69	< 0.01	1.78
5	Xanthine	$[M+H]^+$	153.0392	5.16	0.49	< 0.01	1.07
6	Adenosine monophosphate	$[M+H]^+$	348.0687	7.20	2.14	< 0.01	2.82
7	Famciclovir	$[M+H]^+$	360.1031	2.74	3.61	< 0.01	1.06
8	3-Hydroxy-4-methoxycinnamic acid	$[M+H-H_2O]^+$	177.0531	2.29	0.35	< 0.01	2.59
9	Nicotinyl	$[M+H]^+$	110.0586	0.85	0.26	< 0.01	1.11
10	2,5-Dihydroxycinnamic acid methyl ester	$[M+CH_3COO+2H]^+$	255.0840	0.97	0.48	< 0.01	1.17
11	Anthranilic acid	$[M+H]^+$	138.0536	5.90	0.34	< 0.01	1.74
12	Pyridoxine	$[M+H]^+$	170.0794	1.80	0.34	< 0.01	1.82
13	4-Hydroxyphenylpyruvate	$[M+CH_3COO+2H]^+$	241.0687	0.69	0.45	0.01	1.27
14	L-Arginine	$[M+H]^+$	175.1180	8.88	0.49	0.03	3.62
15	Nicotine	$[M+H]^+$	163.1214	1.27	0.24	0.04	1.02
16	Dimethylglycine	$[M+H]^+$	104.0695	4.25	0.21	0.04	1.58

17	L-Pyroglutamic acid	$[M-H]^-$	128.0349	4.98	0.41	< 0.01	2.50
18	1-Palmitoyl-2-hydroxy-sn-glycero-3-phosphoethanolamine	$[M-H]^-$	452.2783	3.32	0.45	< 0.01	2.28
19	3-Hydorxy-3-methylglutaric acid	$[M-H]^-$	161.0447	6.15	2.01	< 0.01	1.86
20	β-Estradiol 3,17-disulfate	$[M-H]^-$	431.0798	0.40	305.57	< 0.01	8.79
21	UDP-N-acetylglucosamine	$[M-H]^-$	606.0753	7.12	9.09	< 0.01	1.20
22	9-OxoODE	$[M-H]^-$	293.2114	0.62	0.49	< 0.01	1.34
23	PGF3a	$[M-H]^-$	351.2165	1.91	0.34	< 0.01	1.40
24	Scytalone	$[M-H]^-$	193.0502	2.55	0.26	< 0.01	2.15
25	4-Pyridoxic acid	$[M-H]^-$	182.0452	0.69	0.32	< 0.01	1.10
26	Lawsone	$[M-H]^-$	173.0240	1.31	0.25	< 0.01	2.28
27	1,2-Benzenedicarboxylic acid	$[M-H]^-$	165.0187	3.03	3.16	< 0.01	1.39
28	Vanillin	$[M-H]^-$	151.0393	1.18	2.08	0.01	1.39

Note: Differential metabolites were selected using the criteria of a fold change either ≥ 2 or ≤ 0.5, *p* value ≤ 0.05 and VIP score ≥ 1. *m/z*, mass to charge ratio; RT, retention time; BS, black sesame seeds; WS, white sesame seeds; VIP, variable importance in projection.
